# Neutrophil‐to‐lymphocyte and monocyte‐to‐lymphocyte ratios as inflammatory markers in the assessment of glycemic status in diabetic patients of Asir region

**DOI:** 10.1002/ame2.70023

**Published:** 2025-05-07

**Authors:** Ayed A. Dera, Abeer Abdullah Alghamdi, Mesfer Al‐Shahrani, Reem M. Al‐Gahtani, Bashayer Saad Alghamdi, Gaffar Sarwar Zaman, Mohammed Abdul Rasheed, Hassan Al‐Shehri, Lana Al‐qhtani, Syed Parween Ali, Umme Hani, Talha Bin Emran

**Affiliations:** ^1^ Department of Clinical Laboratory Sciences, College of Applied Medical Sciences King Khalid University Abha Saudi Arabia; ^2^ Department of Medical Laboratory King Faisal Medical Complex Taif Saudi Arabia; ^3^ Department of Pathology Dr. VRK Women's Medical College Hyderabad India; ^4^ Asir Central Hospital, Ministry of Health Abha Saudi Arabia; ^5^ Department of Pharmaceutics, College of Pharmacy King Khalid University Abha Saudi Arabia; ^6^ Department of Pharmacy, Faculty of Allied Health Sciences Daffodil International University Dhaka Bangladesh

**Keywords:** diabetes mellitus, glucose metabolism, glycated hemoglobin (HbA1c), inflammatory markers, insulin resistance, neutrophil‐to‐lymphocyte ratio

## Abstract

**Background:**

Diabetes mellitus (DM) is a prevalent chronic metabolic condition characterized by high blood sugar levels, resulting from insufficient insulin production or ineffective insulin use, posing substantial global health issues. Research on the relationship between glycemic status and the ratio of neutrophils to lymphocytes (NLR) and monocytes to lymphocytes (MLR) is limited. This study aimed to fill these knowledge gaps by examining the connection between DM and inflammatory markers within the Asir region.

**Methods:**

Data from 3545 participants were retrospectively analyzed. The dataset, gathered between 2021 and 2023, comprises 38 laboratory tests obtained from the Future Lab Pioneer database. The study's inclusion criteria focused on diabetes profile tests (glycated hemoglobin [HbA1c] and fasting blood glucose [FBG]) and manually computed inflammatory markers (NLR and MLR), which were stratified by age and sex.

**Results:**

This study demonstrated significant differences in NLR levels compared with FBG levels across all adult age groups and adult female participants *(p* < 0.0001), as well as among all elderly age groups (*p* = 0.0006) and elderly women (*p* = 0.01). MLR levels were significant in all adult age groups (*p* = 0.04) and in adult women (*p* = 0.02). When NLR and MLR were compared to HbA1c levels, a significant difference in the mean NLR was found in adult women (*p* = 0.005). Additionally, the mean MLR levels were significant in all adult age groups (*p* = 0.04) and adult women (*p* = 0.02).

**Conclusion:**

Although a larger sample size is necessary for this research, the results indicate that NLR and MLR could serve as valuable indicators for evaluating inflammation in people with disrupted glucose metabolism, particularly in adult and female populations.

## Graphical abstract

Neutrophil‐to‐lymphocyte ratio (NLR) and monocyte‐to‐lymphocyte ratio (MLR) were investigated as potential markers. A total of 3545 subjects were included in the analysis retrospectively. Adult men and women with impaired glucose metabolism were assessed. NLR and MLR may help assess inflammation in individuals with impaired glucose metabolism.

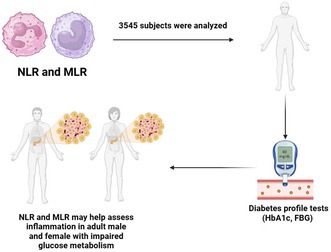

## 1 INTRODUCTION

Diabetes mellitus (DM) is a medical condition characterized by persistently elevated blood glucose levels. There are various types of diabetes, the most common being type 1 diabetes and type 2 diabetes (T2D). In individuals without diabetes, the pancreas releases insulin in response to increasing blood glucose levels, promoting glucose absorption by cells. However, in diabetes, there is a disruption in either the production of insulin by the pancreas or the cellular response to insulin. Type 1 diabetes mellitus (T1D) is an autoimmune disorder in which the body's immune system mistakenly attacks and destroys pancreatic insulin‐producing cells (β cells). This results in insufficient insulin production, which leads to elevated blood glucose levels. Conversely, T2D occurs when cells become resistant to the effects of insulin, a condition known as insulin resistance (IR).^1^

T2D is characterized by persistent chronic inflammation, which plays a pivotal role in its pathophysiology.^2^ Furthermore, microvascular and macrovascular complications associated with T2D, including diabetic nephropathy, proliferative diabetic retinopathy, and diabetic peripheral neuropathy, demonstrate a significant inflammatory burden in their pathogenesis and progression.^3^, ^4^, ^5^ Additionally, inflammatory processes are fundamental characteristics of metabolic disorders frequently comorbid with T2D, particularly metabolic syndrome and obesity, which exhibit complex inflammatory cascades.^6^, ^7^ The neutrophil‐to‐lymphocyte ratio (NLR), a validated marker of systemic inflammation, is elevated across multiple inflammatory conditions, including DM,^5^, ^7^ gastrointestinal pathologies,^8^ cardiovascular disorders,^9^ autoimmune thyroiditis,^10^ various thyroid dysfunctions,^11^ inflammatory bowel disease,^2^ and SARS‐CoV‐2 infection.^12^ The monocyte‐to‐lymphocyte ratio (MLR), another significant inflammatory biomarker, is associated with various pathological conditions, including malignant neoplasms, diabetic nephropathy, functional gastrointestinal disorders, and frailty syndrome.^2^, ^13^, ^14^, ^15^ Due to the inflammatory underpinnings of type 2 DM (T2DM) and its associated complications, along with the established utility of these hematological ratios in inflammatory conditions, investigation of MLR and NLR in the context of T2DM presents a scientifically sound and clinically relevant research direction.

Despite the pancreas producing extra insulin to compensate for IR, blood glucose levels continue to increase because of the cells' reduced responsiveness to insulin. Historically referred to as adult‐onset diabetes, T2D is now increasingly being diagnosed in children and adolescents, primarily due to increasing rates of obesity. Obesity is a significant risk factor for T2D, along with a genetic predisposition. Gestational diabetes is another form of diabetes that occurs during pregnancy and is characterized by elevated blood sugar levels. Although gestational diabetes typically resolves after childbirth, women who have had it are at a higher risk of developing T2D later in life.^16^ Recent studies have focused on understanding the intricate interplay between DM and inflammation, with contributing factors including genetic predisposition, sedentary lifestyle, dietary habits, and tobacco use.^17^, ^18^, ^19^

Research into peripheral blood inflammatory markers, such as NLR and MLR, and pro‐inflammatory cytokines, such as interleukin‐6 (IL‐6) and tumor necrosis factor‐alpha (TNF‐α), has emerged as a key area for understanding the link between DM and inflammation.^20^, ^21^ Elevated levels of these markers have been observed in individuals with DM and are associated with an increased cardiovascular risk, impaired glycemic control, and diabetic complications.^22^ The interplay between IR and inflammation contributes to the cytokine and adipokine surge characteristic of DM pathogenesis,^23^ leading to complications such as vasculopathy, retinopathy, nephropathy, and neuropathy.^24^ Moreover, modulation of immune cells such as T lymphocytes, natural killer cells, natural killer T‐cells (NKT)‐cells, and dendritic cells may contribute to the development and progression of both T1DM and T2DM, although white blood cell (WBC) count has a predictive value for T2DM complications.^25^, ^26^

NLR is a valuable biomarker used in medicine and is a recently identified and readily accessible biomarker of inflammation, reflecting an imbalance in the immune system.^27^ Elevated NLR can indicate various health issues, including inflammation, infection, hematological malignancies, and other medical conditions.^28^ In certain medical conditions, such as DM, chronic kidney disease (CKD), and cardiovascular diseases (CVD), elevated NLR levels are associated with an increased risk of developing complications, including diabetic nephropathy, cardiovascular events, and mortality.^29^ The NLR has been extensively studied in clinical research as a valuable parameter for assessing inflammation and predicting outcomes in various diseases. Its versatility and reliability make it a valuable tool for investigators to explore the pathophysiology and therapeutic interventions for different medical conditions. MLR represents hematological and inflammatory parameters derived from standard blood tests. MLR has been investigated as a potential marker for inflammation and prognostic assessment in various medical conditions.^30^, ^31^ It serves as a cost‐effective and simple indicator of systemic inflammation.

MLR can be readily obtained through routine blood tests conducted under basic laboratory conditions. Elevated MLR in patients undergoing peritoneal dialysis is linked to increased susceptibility to all‐cause mortality.^32^ Incidence of new‐onset CVD events like arrhythmia, coronary heart disease (CAD), heart attack, cardiomyopathy, congenital heart disease and so on. MLR demonstrates promise in forecasting short‐term outcomes in other inflammation‐related disorders, including sepsis, cancer, and CVD.^29^ A heightened MLR may signify an escalated inflammation or dysregulation of the immune system. The NLR serves as a surrogate indicator of inflammation in various cancers,^33^, ^34^ offering a simple, rapid, and cost‐effective means of assessment. Similarly, MLR provides another measure of inflammation by reflecting the release of pro‐inflammatory mediators from monocytes.^35^, ^36^ Furthermore, monocytes play a significant role in inflammation and have been implicated in various inflammatory diseases like atherosclerosis.^37^ Compared to current methods for cancer prognosis, which rely on molecular markers requiring complex and expensive assays, such as immunohistochemistry and quantitative reverse transcription polymerase chain reaction (q‐RT PCR), the measurement of NLR and MLR offers a less‐invasive and more accessible approach. These biomarkers minimize patient discomfort by utilizing peripheral blood samples, while providing valuable insights into different metabolic diseases. Therefore, considerable research has been conducted to leverage NLR and MLR as biomarkers for metabolic disease and cancer prognosis.^38^, ^39^

Understanding the association between DM and inflammatory markers provides insights into the disease mechanisms and offers potential therapeutic avenues. Despite the promising roles of the NLR and MLR in DM diagnosis and management, there is a lack of population‐based studies examining their patterns in relation to glycemic status, particularly in Asian populations. Furthermore, the effect of age on the association between NLR, MLR, and DM warrants further investigation. Therefore, this study aimed to investigate the relationship between DM and inflammatory markers, including NLR and MLR, in the Asir population to enhance our understanding of disease mechanisms and identify potential therapeutic targets.

## 2 METHODOLOGY

### 2.1 Study objectives

The primary aim of this study is to evaluate the relationship between glucose regulation and systemic inflammation, as reflected by NLR and MLR. By analyzing these markers in a diverse sample, this study aims to provide insights into the early inflammatory changes associated with prediabetes and diabetes. Findings from this research will contribute to improved early detection and risk assessment for metabolic disorders in the Saudi population.

### 2.2 Sample size

This study was approved by the Biomedical Ethics Unit of King Khalid University (approval number EMC 2024‐215), Abha, Saudi Arabia. Age, sex, and laboratory data for 3545 subjects with 38 different laboratory tests collected during 2021–2023 were retrieved from the Future Lab Pioneer database and were retrospectively analyzed.

### 2.3 Study design and sample collection

The inclusion criteria were diabetes profile test (glycated hemoglobin [HbA1c], FBG) and inflammatory markers based on age and sex. Inflammatory markers (NLR and MLR) were calculated manually. Records that did not include these variables were excluded from the study. We excluded all pregnant women with anemic diabetes and patients with chronic diseases such as cardiovascular disorders. Gender and age groups were as follows: young (<30 years), adults (31–60 years), and elderly (>60 years). The numbers of participants are presented in Table 1. Normal glucose (NG) was defined as values <110 mg/dL, IFG as 110–125 mg/dL, and hemoglobin (HG) as ≥126 mg/dL. An average HbA1c level of <5.7%, 5.7% to 6.4% indicates prediabetes, and a level of 6.5% or more indicates diabetes; Table 2 presents the total mean of all study subject characteristics. Biochemical parameters such as FBG and HbA1c were determined using the respective kits following the manufacturer's protocols (Roche Diagnostics, USA). The NLR and MLR were derived from a routine complete blood count (CBC) using a CBC analyzer (Beckman) and were manually calculated for each participant using the following formulas:

NLR=neutrophil count/lymphocyte count

MLR=monocyte count/lymphocyte count

**TABLE 1:** Age and gender distribution of study subjects.

Subjects	Number of subjects	NLR mean + SD	MLR mean + SD
Men			
Young	223	1.25 ± 0.68	0.21 ± 0.11
Adult	683	1.37 ± 0.75	0.23 ± 0.11
Elderly	174	1.78 ± 1.11	0.30 ± 0.29
Women			
Young	292	1.36 ± 0.83	0.20 ± 0.03
Adult	784	1.40 ± 0.78	0.21 ± 0.48
Elderly	169	1.48 + 1.19	0.23 + 0.12

Abbreviations: MLR, monocyte‐to‐lymphocyte ratio; NLR, neutrophil‐to‐lymphocyte ratio; SD, standard deviation.

**TABLE 2:** Characteristics of study subjects.

Subjects	Number of subjects	Total HbA1c mean ± SD	Total FBG mean + SD
Men			
Young	223	5.73 ± 1.71	100.96 ± 34.54
Adult	683	6.05 ± 1.25	110.38 ± 32.75
Elderly	174	7.05 ± 1.92	130.59 ± 45.76
Women			
Young	292	5.32 ± 0.69	94.88 ± 17.59
Adult	784	5.96 ± 1.42	109.14 ± 37.65
Elderly	169	5.82 + 1.51	126.44 + 48.18

Abbreviation: FBG, fasting blood glucose; HbA1c, glycated hemoglobin.

### 2.4 Statistical analysis

GraphPad Prism, version 9.2.0 (GraphPad Software, Inc., San Diego, CA, USA), Excel, and SPSS were used for analysis, and statistical significance was set at a *p*‐value of <0.05. Means were compared using one‐way analysis of variance (ANOVA). Association between diabetes profile and inflammatory markers was tested by compared means using one‐way ANOVA. In figures *p*‐values were set as *p < 0.05, **p < 0.01, and ***p < 0.0001.

## 3 RESULTS

### 3.1 NLR exhibited significant differences when analyzed in relation to FBG levels

**FIGURE 1:**
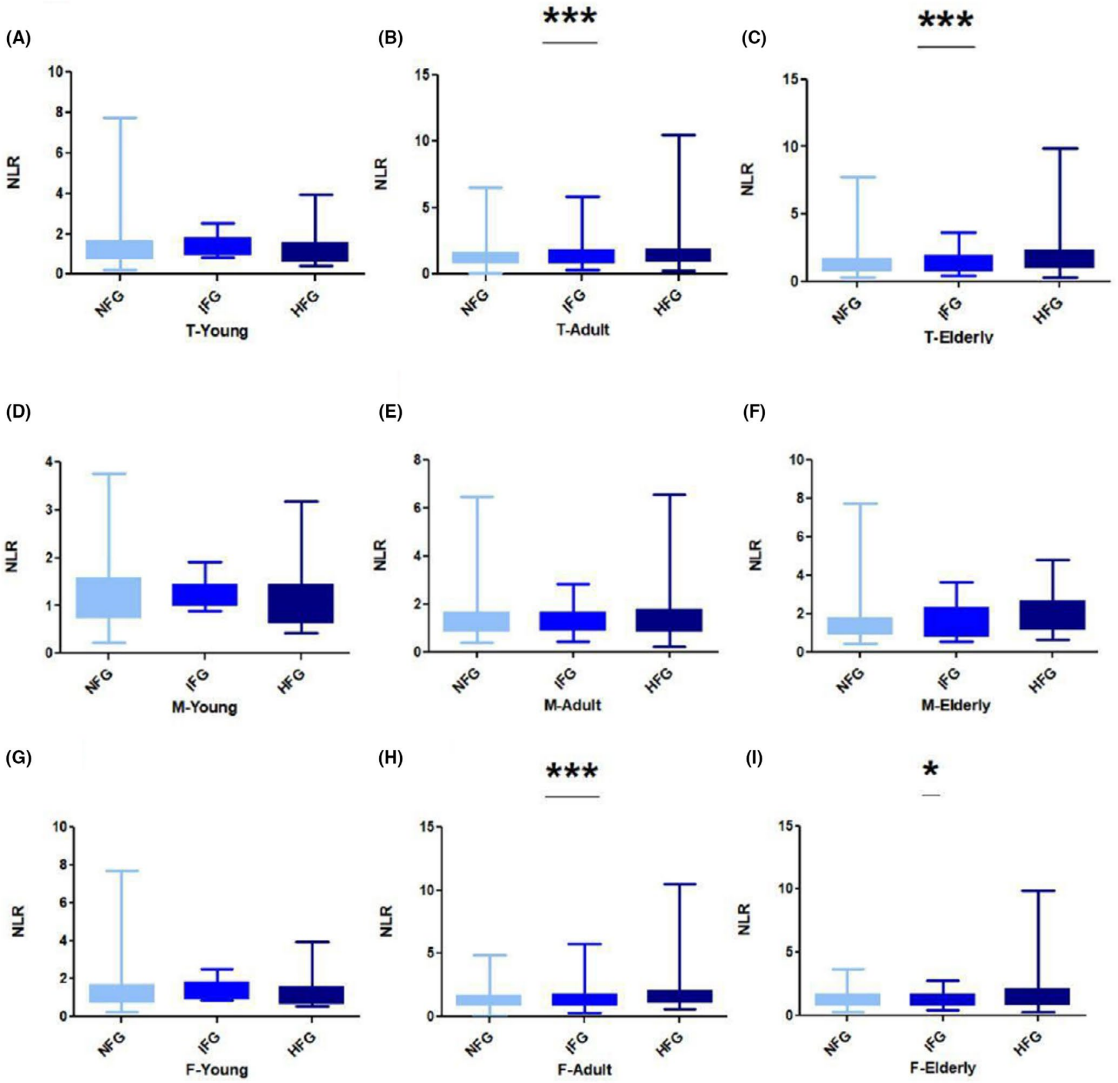
NLR (neutrophil‐to‐lymphocyte ratio) relative to FBG (fasting blood glucose): (A) NLR with FBG in total young population, (B) NLR with FBG in total adult population, (C) NLR with FBG in total elderly population, (D) NLR with FBG in young men, (E) NLR with FBG in adult men, (F) NLR with FBG in elderly men, (G) NLR with FBG in young women, (H) NLR with FBG in adult women, (I) NLR with FBG in elderly women. *p < 0.05, **p < 0.01, and ***p < 0.0001.

The mean NLR levels were significantly different across age groups based on gender, total adult FBG level (1.40 ± 0.69 and 1.62 ± 1.13 vs. 1.34 ± 0.67, ANOVA *p* < 0.0001) (Figure 1B), and total elderly population (1.41 ± 0.71 and 1.93 ± 1.41 vs. 1.46 ± 0.99, ANOVA *p* = 0.0006) (Figure 1C). When the IFG and HG groups were compared with the NG group retrospectively, the mean NLR levels were significantly increased in adult women with elevated FBG (1.44 ± 0.78 and 1.18 ± 1.37 vs. 1.33 ± 0.60, ANOVA *p* < 0.0001) (Figure 1H) and elderly women (1.29 ± 0.56 and 1.86 ± 1.81 vs. 1.30 ± 0.67 *p* = 0.01) (Figure 1I). When the IFG and HG groups were compared with the NG group retrospectively, the mean NLR was not significantly different across all young age groups (Figure 1A), young men (Figure 1D), adult men (Figure 1E), elderly men (Figure 1F), and young women (Figure 1G). A comparison of all the NLR means is presented in Table 3.

**TABLE 3:** Characteristics of study subjects relative to NLR in different age groups and genders.

Variables	Normal mean ± SD	Pre‐diabetic mean ± SD	Diabetic mean ± SD	*p*‐Value
NLR				
Total young FBG	1.29 ± 0.74	1.36 ± 0.46	1.28 ± 0.83	0.93
Total adult FBG	1.34 ± 0.67	1.40 ± 0.69	1.62 ± 1.13	<0.0001
Total elderly FBG in men	1.46 ± 0.99	1.41 ± 0.71	1.93 ± 1.41	0.0006
Male young FBG	1.42 ± 0.63	1.26 ± 0.30	1.21 ± 0.66	0.9
Male adult FBG	1.35 ± 0.76	1.35 ± 0.57	1.45 ± 0.82	0.4
Male elderly FBG	1.60 ± 1.19	1.63 ± 0.85	1.96 ± 1.00	0.1
Female young FBG	1.33 ± 0.80	1.40 ± 0.50	1.38 ± 0.94	0.9
Female adult FBG	1.33 ± 0.60	1.44 ± 0.78	1.18 ± 1.37	<0.0001
Female elderly total FBG	1.30 ± 0.67	1.29 ± 0.56	1.86 ± 1.81	0.01
Total young HbA1c	1.34 ± 0.70	1.24 ± 0.71	1.25 ± 0.80	0.33
Total adult HbA1c	1.36 ± 0.73	1.36 ± 0.67	0.50 ± 0.92	0.07
Total elderly HbA1c	1.64 ± 1.18	1.63 ± 1.35	1.60 ± 1.00	0.9
Male young HbA1c	1.30 ± 0.61	1.21 ± 0.76	1.22 ± 0.73	0.7
Male adult HbA1c	1.35 ± 0.71	1.39 ± 0.71	1.36 ± 0.60	0.8
Male elderly HbA1c	1.76 ± 1.34	1.63 ± 1.20	1.74 ± 0.87	0.8
Female young HbA1c	1.37 ± 0.74	1.27 ± 0.64	1.31 ± 0.80	0.7
Female adult HbA1c	1.38 ± 0.74	1.33 ± 0.62	1.64 ± 1.15	0.005
Female elderly HbA1c	1.44 ± 0.82	1.60 ± 1.43	1.43 ± 1.10	0.7

Abbreviations: FBG, fasting blood glucose; HbA1c, glycated hemoglobin; NLR, neutrophil‐to‐lymphocyte ratio.

### 3.2 NLR exhibited significant differences when analyzed in relation to HbA1c levels

**FIGURE 2:**
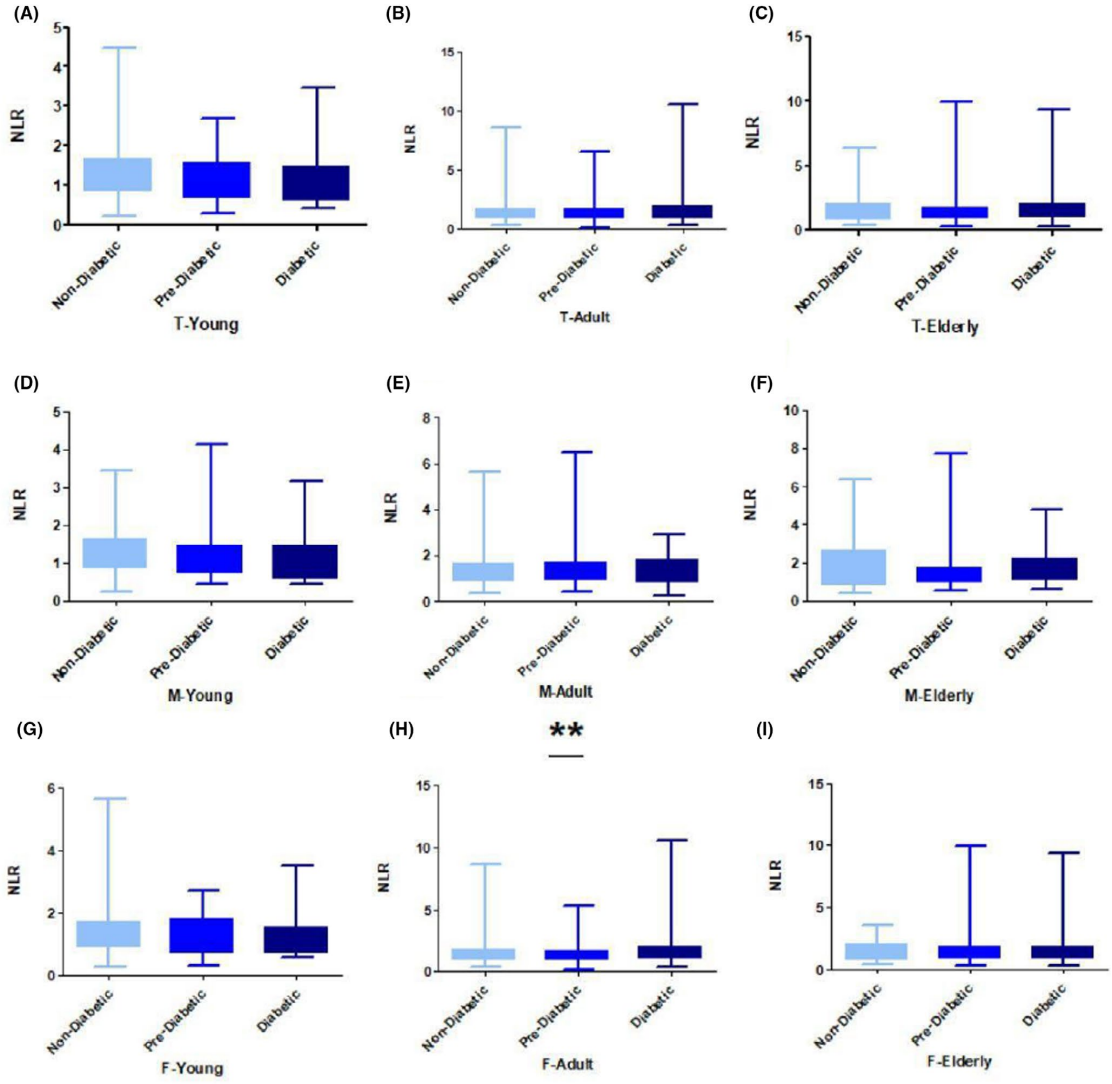
NLR (neutrophil‐to‐lymphocyte ratio) relative to HbA1c (glycated hemoglobin): (A) NLR with HbA1c in total young, (B) NLR with HbA1c in total adult, (C) NLR with HbA1c in total elderly, (D) NLR with HbA1c in young men, (E) NLR with HbA1c in adult men, (F) NLR with HbA1c in elderly men, (G) NLR with HbA1c in young women, (H) NLR with HbA1c in adult women, and (I) NLR with HbA1c in elderly women. *p < 0.05, **p < 0.01, and ***p < 0.0001.

The mean NLR levels were significantly different across the adult age group in women with different HbA1c levels (1.33 ± 0.62 and 1.64 ± 1.15 vs. 1.38 ± 0.74 *p* = 0.005) (Figure 2H) when prediabetic and diabetic groups were compared with regular groups retrospectively. The mean NLR level exhibited no significance across all young age groups (Figure 2A), total adult (Figure 2B), total elderly (Figure 2C), young men (Figure 2D), adult men (Figure 2E), elderly men (Figure 2F), young women (Figure 2G), and elderly women (Figure 2I). A comparison of all the NLR means is presented in Table 3.

### 3.3 MLR exhibited significant differences when analyzed in relation to FBG levels

**FIGURE 3:**
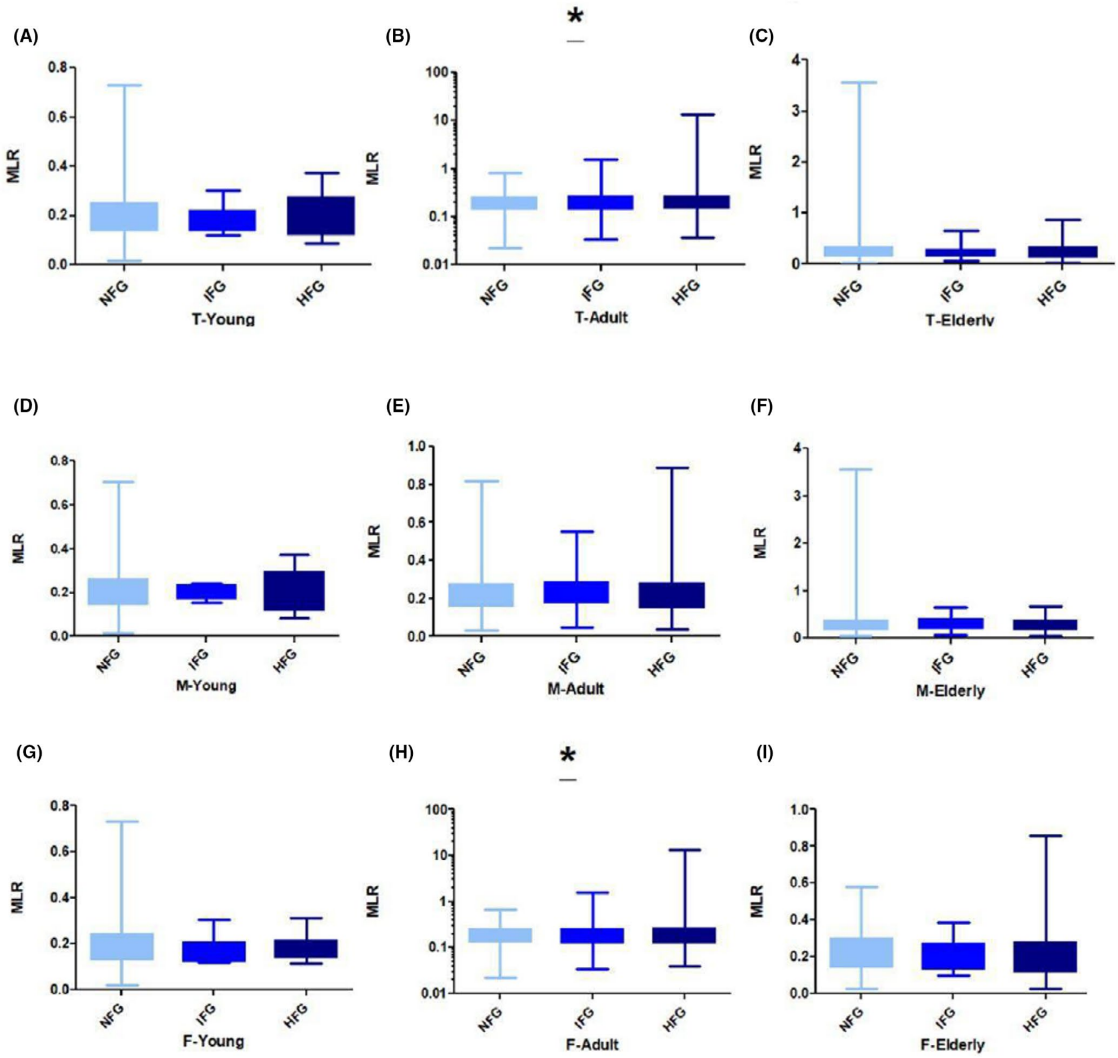
MLR (monocyte‐to‐lymphocyte ratio) relative to FBG (fasting blood glucose): (A) MLR with FBG in total young, (B) MLR with FBG in total adults, (C) MLR with FBG in total elderly, (D) MLR with FBG in young men, (E) MLR with FBG in adult men, (F) MLR with FBG in elderly men, (G) MLR with FBG in young women, (H) MLR with FBG in adult women, and (I) MLR with FBG in elderly women. *p < 0.05, **p < 0.01, and ***p < 0.0001.

The mean MLR levels were significant across age groups based on gender in total adults (1.22 ± 0.14 and 0.28 ± 0.93 vs. 0.21 ± 0.10, ANOVA *p* = 0.04) (Figure 3B), and adult women (0.20 ± 0.17 and 0.34 ± 1.36 vs. 0.19 ± 0.09, ANOVA *p* = 0.02) (Figure 3H) when IFG and HG groups were compared with NG group retrospectively. The mean MLR level was not significantly different across all young age groups (Figure 3A), the total elderly population (Figure 3C), young men (Figure 3D), adult men (Figure 3E), elderly men (Figure 3F), young women (Figure 3G), and elderly women (Figure 3I). A comparison of all the MLR means is presented in Table 4.

**TABLE 4:** Characteristics of study subjects relative to MLR across different age groups and genders.

Variables	Normal mean ± SD	Prediabetic mean ± SD	Diabetic mean ± SD	*p*‐Value
MLR				
Total young FBG	0.20 ± 0.09	0.18 ± 0.05	0.20 ± 0.08	0.7
Total adult FBG	0.21 ± 0.10	1.22 ± 0.14	0.28 ± 0.93	0.04
Total elderly	0.28 ± 0.29	0.23 ± 0.11	0.52 ± 0.15	0.4
FBG				
Male young FBG	0.20 ± 0.10	0.19 ± 0.03	0.21 ± 0.09	0.9
Male adult FBG	0.22 ± 0.10	0.23 ± 0.09	0.22 ± 0.11	0.6
Male elderly FBG	0.32 ± 0.39	0.30 ± 0.14	0.27 ± 0.14	0.5
Female young FBG	0.19 ± 0.09	0.17 ± 0.05	0.19 ± 0.05	0.7
Female adult FBG	0.19 ± 0.09	0.20 ± 0.17	0.34 ± 1.36	0.02
Female elderly FBG	0.23 ± 0.11	0.20 ± 0.07	0.22 ± 0.16	0.4
Total young HbA1c	0.206 ± 0.10	0.18 ± 0.10	0.208 ± 0.10	0.5
Total adult HbA1c	0.20 ± 0.09	0.21 ± 0.10	0.28 ± 0.93	0.04
Total elderly HbA1c	0.262 ± 0.15	0.269 ± 0.36	0.24 ± 0.14	0.73
Male young HbA1c	0.21 ± 0.09	0.19 ± 0.12	0.22 ± 0.11	0.6
Male adult HbA1c	0.21 ± 0.09	0.24 ± 0.11	0.22 ± 0.07	0.09
Male elderly HbA1c	0.29 ± 0.16	0.33 ± 0.51	0.27 ± 0.16	0.5
Female young HbA1c	0.20 ± 0.10	0.18 ± 0.07	0.18 ± 0.69	0.6
Female adult HbA1c	0.19 ± 0.09	0.19 ± 0.08	0.35 ± 0.34	0.02
Female elderly HbA1c	0.22 ± 0.11	0.21 ± 0.11	0.20 ± 0.11	0.8

Abbreviations: FBG, fasting blood glucose; HbA1c, glycated hemoglobin; MLR, monocyte‐to‐lymphocyte ratio.

### 3.4 MLR exhibited significant differences when analyzed in relation to HbA1c levels

**FIGURE 4:**
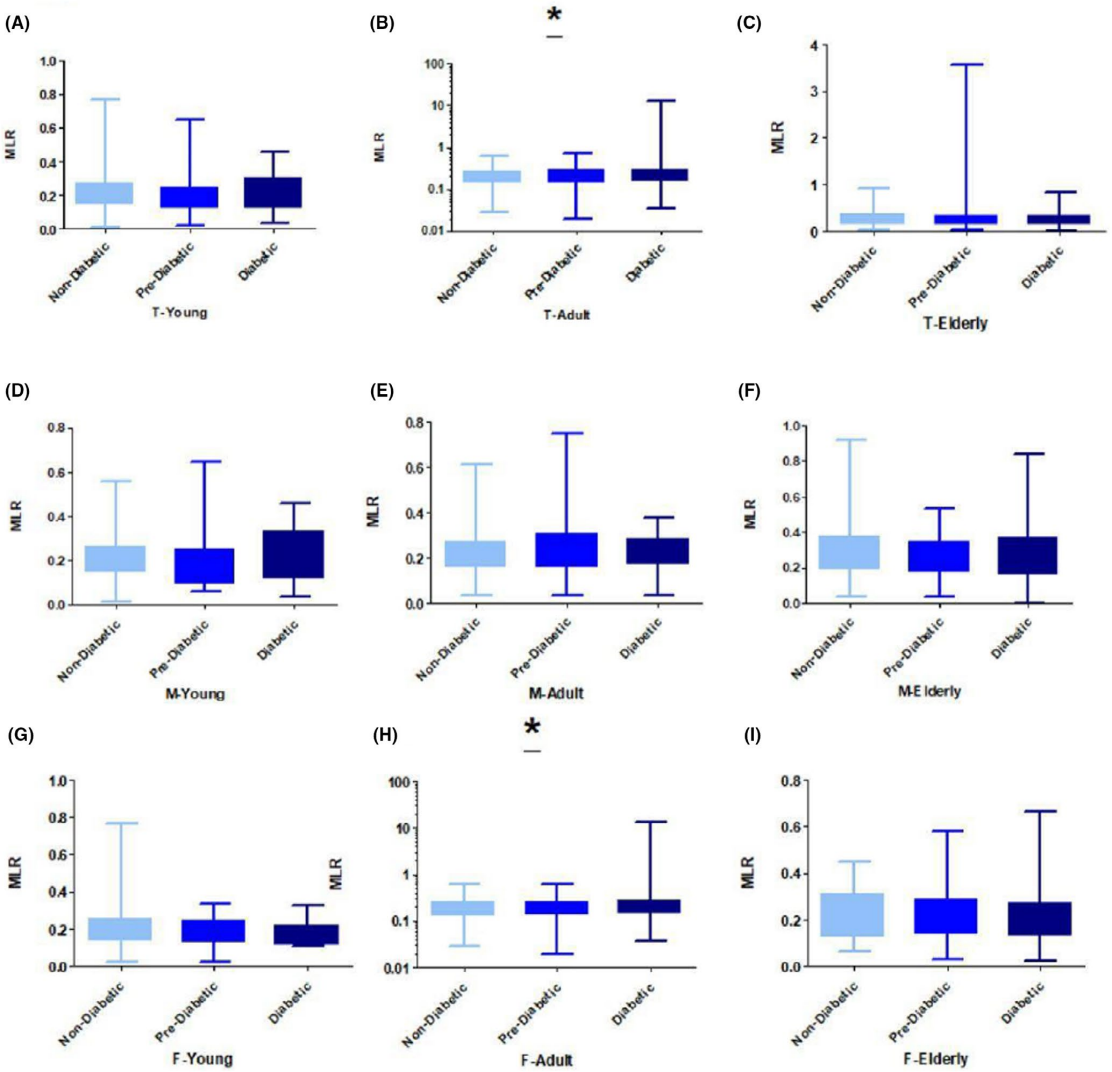
MLR (monocyte‐to‐lymphocyte ratio) relative to HbA1c (glycated hemoglobin): (A) MLR with HbA1c in total young, (B) MLR with HbA1c in total adult, (C) MLR with HbA1c in total elderly, (D) MLR with HbA1c in young men, (E) MLR with HbA1c in adult men, (F) MLR with HbA1c in elderly men, (G) MLR with HbA1c in young women, (H) MLR with HbA1c in adult women, and (I) MLR with HbA1c in elderly women. *p < 0.05, **p < 0.01, and ***p < 0.0001.

The mean MLR levels were significant across age groups based on gender in total adults (0.21 ± 0.10 and 0.28 ± 0.93 vs. 0.20 ± 0.09 *p* = 0.04) (Figure 4B) when prediabetic and diabetic groups were compared with normal groups retrospectively. The mean MLR level was significantly increased in adult women (0.35 ± 0.34 vs. 0.19 ± 0.09, ANOVA *p* = 0.02) (Figure 4H) when the diabetic group was compared with the nondiabetic normal group. The mean MLR level was not significantly different across all young age groups (Figure 4A), total elderly (Figure 4C), young men (Figure 4D), adult men (Figure 4E), elderly men (Figure 4F), young women (Figure 4G), and elderly women (Figure 4I). A comparison of all the MLR means is presented in Table 4.

## 4 DISCUSSION

Current research demonstrates that individuals with diabetes and prediabetes consistently exhibit higher levels of NLR and MLR across various age groups and sexes compared to those without diabetes. We found a significant association between NLR, MLR, FBG, and HbA1c levels among different age categories based on sex, suggesting a potential link between elevated FBG and HbA1c levels and systemic inflammation, as indicated by increased NLR and MLR. These findings shed light on possible immune responses to glucose dysregulation and provide insights into the potential impact of prolonged glucose control on systemic inflammation.

Several studies have highlighted a significant correlation between NLR and diabetes.^38^, ^39^ Shiny et al.^40^ and Lou et al.^41^ have both emphasized a connection between elevated NLR and conditions such as glucose intolerance and IR in individuals with T2D. Research on microvascular complications associated with diabetes has suggested that NLR can predict the early onset of retinopathy,^42^ diabetic foot ulcers,^43^ and diabetic nephropathy. Moreover, it has been linked to increased carotid artery intima‐media thickness in individuals with T2D.^44^ Sefil et al.^45^ suggested a potential link between an increased NLR and elevated HbA1c levels in patients with T2D. Shiny et al.^40^ study explored the relationship between the NLR and the occurrence of DM and impaired glucose tolerance, revealing a significant association between these factors.^41^ Oh et al.^46^ and Akin et al.^47^ established a connection between glycemic control and hematological indices in individuals with T2D, suggesting that the NLR may serve as a marker for diabetic management and complications. Previous studies have also established an association between an elevated NLR and increased HbA1c levels in individuals with T2DM.^48^ Hyperglycemia may impair lymphocyte function and reduce lymphocyte counts, contributing to an elevated NLR and MLR.^49^ Further research is needed on how glucose levels specifically affect different lymphocyte subsets.

It has been demonstrated that in patients with T2DM, the expression of activation markers on neutrophil membranes differs from that in healthy controls. This is evidenced by decreased expression of the adhesion molecule lymphocyte function associated antigen 3 (LFA‐3), increased levels of activation markers such as CD11B and CD66B, and increased adhesion of neutrophils to endothelial cells, leading to systemic inflammation and endothelial damage.^50^, ^51^, ^52^ In preclinical models, research has demonstrated that neutrophils can induce the release of IL‐1β and neutrophil elastase through the NF‐κB pathway, disrupting insulin signaling and degrading insulin receptor substrate‐1, respectively, thereby contributing to IR.^53^, ^54^ Th17 cells represent a subset of CD4 + T cells that are characterized by the secretion of pro‐inflammatory factors, including IL‐17. Studies have indicated that IL‐17 stimulates the production of TNF‐α and is implicated in the development of IR.^55^ Regulatory T (Treg) cells constitute a minor subset of T lymphocytes known for their pivotal role in suppressing inflammatory responses.^56^ During the progression of diabetes, Treg cells can inhibit Th1 and Th17 cell responses through the regulation of the microenvironment and alteration of surface receptor expression, consequently ameliorating IR.^57^ Nonetheless, patients with diabetes exhibit a significant reduction in the number of Treg cells.^58^

Monocytes are markers of chronic inflammation associated with increased levels of adipose tissue. They produce pro‐inflammatory cytokines, and their levels are correlated with atherogenesis and IR in obese individuals.^59^ Huang et al.^60^ observed a correlation between MLR and diabetic retinopathy, suggesting that MLR is a risk factor for diabetes. Grossman et al.^61^ reported elevated levels of WBCs, granulocytes, and monocytes in normoglycemic individuals compared with those with diabetes. MLR exhibited greater stability due to the equilibrium between monocyte and lymphocyte levels. MLR has emerged as a cost‐effective inflammatory biomarker, with Huang et al.^60^ showing higher MLRs in individuals with diabetic retinopathy, indicating that MLR is a predictor of its onset.

The observation that individuals with prediabetes and diabetes have higher NLR and MLR levels compared to nondiabetic individuals suggests an association between elevated FBG and HbA1c levels and systemic inflammation in adults and elderly women. This indicates a dose–response relationship between long‐term glucose control and inflammation, possibly due to metabolic inflammation associated with obesity, IR, and diabetes. Tanaka et al.^62^ showed that T lymphocytes and their subsets were characteristically reduced in obese individuals. Worsening obesity might increase lymphopenia development, which, in turn, increases the NLR. Chronic hyperglycemia and IR disrupt immune function, leading to an inflammatory state characterized by increased NLR and MLR; however, the exact mechanisms have not been fully elucidated.^49^

Previous research by Wellen and Hotamisligil has emphasized the significance of inflammation in diabetes and obesity, with NLR serving as a marker for evaluating glycemic control alongside HbA1c.^63^ An elevated NLR may indicate increased neutrophil levels and decreased lymphocyte count. Hyperglycemia or advanced glycated end products trigger sustained neutrophil activation, as demonstrated by elevated neutrophil alkaline phosphatase activity, but their effects on neutrophil, lymphocyte, and monocyte populations are not well characterized.^64^ This can result in higher neutrophil counts and NLR values. However, the molecular pathways mediating this effect require further investigation. Consequently, neutrophils in diabetic individuals exhibit increased necrosis, heightened reactive oxygen species production, and notably reduced chemotactic responses.^65^

Elderly individuals with high FBG levels exhibit higher NLR than those with normal FBG levels, whereas no significant differences were observed in MLR among different age groups, suggesting that factors beyond FBG may influence MLR in these age groups. This underscores the potential influence of age‐related factors, such as inflammation and chronic low‐grade inflammation in older individuals, on immune response. Aging‐related alterations in immune function, including changes in neutrophil and lymphocyte populations, may contribute to variations in NLR. MLR, influenced by factors beyond FBG levels, could reflect age‐related shifts in monocyte function and distribution and other comorbidities, such as CVD and CKD.

Franceschi et al. explored inflammation and its relevance in age‐related diseases, emphasizing the role of chronic inflammation in aging and associated ailments.^66^ The absence of significant differences in NLR and MLR among the young and elderly age groups when categorized by HbA1c levels suggests that factors beyond long‐term glucose control may impact NLR and MLR levels.^67^ Aging‐related alterations in immune function and chronic low‐grade inflammation may interact with glucose dysregulation to influence NLR and MLR levels in complex distinct ways that are not fully understood, especially in young and elderly individuals. Clinicians should consider various factors affecting HbA1c levels, such as genetic, hematologic, and illness‐related factors, when assessing long‐term glycemic control in patients with DM. Hemoglobinopathies, anemia, and disorders associated with accelerated red cell turnover, such as malaria, are the most common important factors affecting HbA1c levels worldwide.^68^ Furthermore, recent blood transfusion, use of erythropoiesis‐stimulating drugs, end‐stage kidney disease, and pregnancy may cause discrepancies between the HbA1c result and the patient's true mean glycemia.^69^

In our study, we observed that women with DM exhibit a higher tendency to express elevated NLR and MLR levels than men; however, the underlying mechanisms, such as hormonal factors, require further investigation. This finding suggests that several factors may contribute to an increased risk of DM in women. It was perceived that hormonal fluctuations, particularly during puberty, pregnancy, and menopause, affect insulin sensitivity and glucose metabolism.^70^ Additionally, lifestyle factors such as diet quality and physical activity levels play significant roles in the development of diabetes.^1^ Furthermore, obesity, which is more prevalent among women than among men, is a major risk factor for T2D.^71^ Psychosocial stress, which may be experienced at higher levels by women, can also contribute to the development of diabetes through various pathways, including hormonal imbalances and changes in lifestyle behaviors.^72^

## 5 LIMITATION

The study was conducted in the Asian region, and the patients in the present study were of Saudi origin. Larger studies are required to determine whether the observations in this study are applicable to individuals of various races and ethnicities. The present study was observational, and further studies on clinical outcomes should be conducted. Moreover, the study cannot address the prognostic and diagnostic roles of NLR and MLR in the glycemic control of T2DM patients and requires more rigorous clinical validation. Clinical factors such as the association of body mass index, duration of diabetes, other comorbidities, hormonal influences, lifestyle factors, genetic factors, and medication use linking glycemic status to NLR and MLR have not been elucidated, which highlights the research gap in understanding the mechanism. Larger studies across diverse populations are needed to determine whether the observed relationships between glycemic markers and NLR/MLR are consistent across different ethnicities and races.

## 6 CONCLUSION

The current study showed that the mean NLR levels were significantly different in relation to FBG levels among all adult age groups and adult women (*p <* 0.0001). Similarly, among the total elderly age groups (*p* = 0.0006) and elderly women (*p* < 0.01), MLR levels were significant across all adult age groups (*p* = 0.04) and adult women (*p* = 0.02). When NLR and MLR were assessed in relation to HbA1c levels, a significant difference in the mean NLR was observed in adult women (*p* = 0.005). In addition, the mean MLR levels were significant among all adult age groups (*p* = 0.04) and adult women (*p* = 0.02). These findings suggest that NLR and MLR are valuable markers for assessing inflammation in individuals with impaired glucose metabolism, especially in adults and women. Although associations between glycemic status and NLR/MLR have been observed, significant gaps persist in elucidating the precise biological mechanisms and clinical implications of these relationships.

## AUTHOR CONTRIBUTIONS

**Ayed A. Dera:** Supervision; validation. **Abeer Abdullah Alghamdi:** Data curation; validation. **Mesfer Al‐Shahrani:** Conceptualization; project administration. **Reem M. Al‐Gahtani:** Investigation; resources; supervision. **Bashayer Saad Algamdi:** Investigation; writing – review and editing. **Gaffar Sarwar Zamam:** Data curation; project administration; resources. **Mohammed Abdul Rasheed:** Formal analysis; supervision; validation. **Hassan Al‐Shehri:** Investigation; resources; writing – review and editing. **Lana Al‐qhtani:** Conceptualization; software; writing – review and editing. **Syed Parween Ali:** Formal analysis; resources; writing – original draft. **Umme Hani:** Data curation; methodology; supervision. **Talha Bin Emran:** Conceptualization; methodology; supervision.

## FUNDING INFORMATION

None.

## CONFLICT OF INTEREST STATEMENT

None.

## ETHICS STATEMENT

This study was approved by the Biomedical Ethics Unit of King Khalid University (approval number EMC 2024‐215), Abha, Saudi Arabia.

## Data Availability

None.

## References

[1] Rooney MR , Tang O , Echouffo Tcheugui JB , et al. American Diabetes Association framework for glycemic control in older adults: implications for risk of hospitalization and mortality. Diabetes Care. 2021;44(7):1524‐1531. doi:10.2337/DC20-3045 34006566 PMC8323179

[2] Aktas G , Duman TT , Atak BM , et al. Irritable bowel syndrome is associated with novel inflammatory markers derived from hemogram parameters. Fam Med Prim Care Rev. 2020;22(2):107‐110. doi:10.5114/fmpcr.2020.95311

[3] Tel BMA , Tel MR , Bilgin S , Duman TT , Aktas G . Diagnostic value of HALP score in detecting diabetic nephropathy in patients with type 2 diabetes mellitus. Ibnosina J Med Biomed Sci. 2024;16(3):116‐122. doi:10.1055/s-0044-1787998

[4] Aktas G . Serum C‐reactive protein to albumin ratio as a reliable marker of diabetic neuropathy in type 2 diabetes mellitus. Biomol Biomed. 2024;24(5):1380‐1386. doi:10.17305/bb.2024.10426 38635449 PMC11379001

[5] Aktas G . Association between the prognostic nutritional index and chronic microvascular complications in patients with type 2 diabetes mellitus. J Clin Med. 2023;12(18):5952. doi:10.3390/jcm12185952 37762893 PMC10531521

[6] Dedemen B , Duman TT , Dedemen MM , Aktas G . Effect of sodium glucose Co‐transporter 2 inhibitor use on anthropometric measurements and blood glucose in obese and non‐obese type 2 diabetic patients. Clin Nutr ESPEN. 2024;63:515‐519. doi:10.1016/j.clnesp.2024.07.016 39047870

[7] Basaran E , Aktas G . The relationship of vitamin D levels with hemogram indices and metabolic parameters in patients with type 2 diabetes mellitus. AIMS Med Sci. 2024;11(1):47‐57. doi:10.3934/medsci.2024004

[8] Aktas G , Tel BA , Duman TT . Inflammatory indices derived from hemogram as prognostic markers of cardiovascular outcome. Int J Cardiovasc Sci. 2024;37:e20240012. doi:10.36660/ijcs.20240012

[9] Sahin S , Sarikaya S , Alcelik A , et al. Neutrophil to lymphocyte ratio is a useful predictor of atrial fibrillation in patients with diabetes mellitus. Acta Med Mediterr. 2013;29(4):847‐851.

[10] Aktas G , Sit M , Dikbas O , et al. Elevated neutrophil‐to‐lymphocyte ratio in the diagnosis of Hashimoto's thyroiditis. Rev Assoc Med Bras. 2017;63(12):1065‐1068. doi:10.1590/1806-9282.63.12.1065 29489971

[11] Afsin H , Aktas G . Platelet to lymphocyte and neutrophil to lymphocyte ratios are useful in differentiation of thyroid conditions with normal and increased uptake. Ethiop J Health Dev. 2021;35:1‐5.

[12] Aktas G . Hematological predictors of novel coronavirus infection. Rev Assoc Med Bras. 2021;67:1‐2. doi:10.1590/1806-9282.67.SUPPL1.20200678 34259763

[13] Catal O , Ozer B , Sit M , Aktas G , Erkol H . The role of monocyte to lymphocyt ratio in predicting metastasis in rectal cancer. Ann Med Res. 2021;28(3):527. doi:10.5455/annalsmedres.2020.05.466

[14] Kocak MZ , Aktas G , Duman TT , et al. Monocyte lymphocyte ratio As a predictor of diabetic kidney injury in type 2 diabetes mellitus; the MADKID study. J Diabetes Metab Disord. 2020;19(2):997‐1002. doi:10.1007/s40200-020-00595-0 33553019 PMC7843868

[15] Tel BMA , Bilgin S , Kurtkulagi O , et al. Frailty in Diabetic Subjects during COVID‐19 and Its Association with HbA1c, Mean Platelet Volume and Monocyte/Lymphocyte Ratio. Clin Diabetol. 2022;11(2):119‐126. doi:10.5603/DK.a2022.0015

[16] Adam S , McIntyre HD , Tsoi KY , et al. Pregnancy as an opportunity to prevent type 2 diabetes mellitus: FIGO best practice advice. Int J Gynecol Obstet. 2023;160(S1):56‐67. doi:10.1002/ijgo.14537 PMC10107137 36635082

[17] Al‐Rubeaan K , Siddiqui K , Saeb ATM , Nazir N , Al‐Naqeb D , Al‐Qasim S . ACE I/D and MTHFR C677T polymorphisms are significantly associated with type 2 diabetes in Arab ethnicity: a meta‐analysis. Gene. 2013;520(2):166‐177. doi:10.1016/j.gene.2013.02.017 23458876

[18] Al‐Nozha MM , Al‐Mazrou YY , Arafah MR , et al. Smoking in Saudi Arabia and its relation to coronary artery disease. J Saudi Heart Assoc. 2009;21(3):169‐176. doi:10.1016/j.jsha.2009.06.007 23960568 PMC3727358

[19] Mahfouz AA , Abdelmoneim I , Khan MY , et al. Obesity and related behaviors among adolescent school boys in Abha City, Southwestern Saudi Arabia. J Trop Pediatr. 2008;54(2):120‐124. doi:10.1093/tropej/fmm089 18039676

[20] Huang J , Xiao Y , Xu A , Zhou Z . Neutrophils in type 1 diabetes. J Diabetes Investig. 2016;7(5):652‐663. doi:10.1111/jdi.12469 PMC5009125 27181374

[21] Mangaonkar AA , Tande AJ , Bekele DI . Differential diagnosis and workup of Monocytosis: a systematic approach to a common hematologic finding. Curr Hematol Malig Rep. 2021;16(3):267‐275. doi:10.1007/s11899-021-00618-4 33880680 PMC8057007

[22] Bhat T , Teli S , Rijal J , et al. Neutrophil to lymphocyte ratio and cardiovascular diseases: a review. Expert Rev Cardiovasc Ther. 2013;11(1):55‐59. doi:10.1586/erc.12.159 23259445

[23] Goldberg RB . Cytokine and cytokine‐like inflammation markers, endothelial dysfunction, and imbalanced coagulation in development of diabetes and its complications. J Clin Endocrinol Metab. 2009;94(9):3171‐3182. doi:10.1210/jc.2008-2534 19509100

[24] Bailes BK . Diabetes mellitus and its chronic complications. AORN J. 2002;76(2):265‐274. doi:10.1016/S0001-2092(06)61065-X 12194653

[25] Daryabor G , Atashzar MR , Kabelitz D , Meri S , Kalantar K . The effects of type 2 diabetes mellitus on organ metabolism and the immune system. Front Immunol. 2020;11:1582. doi:10.3389/fimmu.2020.01582 32793223 PMC7387426

[26] Tang L , Wang H , Cao K , et al. Dysfunction of circulating CD3+CD56+ NKT‐like cells in type 2 diabetes mellitus. Int J Med Sci. 2023;20(5):652‐662. doi:10.7150/ijms.83317 37082729 PMC10110473

[27] Shi C , Pamer EG . Monocyte recruitment during infection and inflammation. Nat Rev Immunol. 2011;11(11):762‐774. doi:10.1038/nri3070 21984070 PMC3947780

[28] Shi L , Qin X , Wang H , et al. Elevated neutrophil‐to‐lymphocyte ratio and monocyte‐tolymphocyte ratio and decreased platelet‐to‐lymphocyte ratio are associated with poor prognosis in multiple myeloma. Oncotarget. 2017;8(12):18792‐18801. doi:10.18632/oncotarget.13320 27852046 PMC5386647

[29] Luo X , Wan D , Xia R , Liao R , Su B . Prognostic value of the baseline and early changes in monocyte‐to‐lymphocyte ratio for short‐term mortality among critically ill patients with acute kidney injury. J Clin Med. 2023;12(23):7353. doi:10.3390/jcm12237353 38068405 PMC10707087

[30] Van Oostrom AJHHM , Sijmonsma TP , Verseyden C , et al. Postprandial recruitment of neutrophils may contribute to endothelial dysfunction. J Lipid Res. 2003;44(3):576‐583. doi:10.1194/jlr.M200419-JLR200 12562833

[31] Berliner S , Rogowski O , Rotstein R , et al. Activated polymorphonuclear leukocytes and monocytes in the peripheral blood of patients with ischemic heart and brain conditions correspond to the presence of multiple risk factors for atherothrombosis. Cardiology. 2000;94(1):19‐25. doi:10.1159/000007041 11111140

[32] Yang Y , Xu Y , Lu P , Zhou H , Yang M , Xiang L . The prognostic value of monocyte‐to‐lymphocyte ratio in peritoneal dialysis patients. Eur J Med Res. 2023;28(1):152. doi:10.1186/s40001-023-01073-y 37038225 PMC10084613

[33] Gao Y , Wang WJ , Zhi Q , et al. Neutrophil/lymphocyte ratio is a more sensitive systemic inflammatory response biomarker than platelet/lymphocyte ratio in the prognosis evaluation of unresectable pancreatic cancer. Oncotarget. 2017;8(51):88835‐88844. doi:10.18632/oncotarget.21340 29179480 PMC5687650

[34] Akinci Ozyurek B , Erdogan Y , Yilmaz Demirci N , Buyukyaylaci Ozden S , Sahin Ozdemirel T . Our experience with pirfenidone in patients with idiopathic pulmonary fibrosis. Eur J Pulmonol. 2016;18(1):61‐62. doi:10.5152/ejp.2016.96268

[35] Krenn‐Pilko S , Langsenlehner U , Thurner EM , et al. The elevated preoperative platelet‐to‐lymphocyte ratio predicts poor prognosis in breast cancer patients. Br J Cancer. 2014;110(10):2524‐2530. doi:10.1038/bjc.2014.163 24675383 PMC4021515

[36] Yan MJ , Jurasz P . The role of platelets in the tumor microenvironment: from solid tumors to leukemia. Biochim Biophys Acta, Mol Cell Res. 2016;1863(3):392‐400. doi:10.1016/j.bbamcr.2015.07.008 26193075

[37] Jaipersad AS , Lip GYH , Silverman S , Shantsila E . The role of monocytes in angiogenesis and atherosclerosis. J Am Coll Cardiol. 2014;63(1):1‐11. doi:10.1016/j.jacc.2013.09.019 24140662

[38] Sun X , Cui F , Yin H , et al. Association between EGFR mutation and expression of BRCA1 and RAP80 in non‐small cell lung cancer. Oncol Lett. 2018;16(2):2201‐2206. doi:10.3892/ol.2018.8938 30008919 PMC6036334

[39] Brennan DJ , Gallagher WM . Prognostic ability of a panel of immunohistochemistry markers ‐ retailoring of an “old solution”. Breast Cancer Res. 2008;10(1):102. doi:10.1186/bcr1854 18331621 PMC2374964

[40] Shiny A , Bibin YS , Shanthirani CS , et al. Association of neutrophil‐lymphocyte ratio with glucose intolerance: an indicator of systemic inflammation in patients with type 2 diabetes. Diabetes Technol Ther. 2014;16(8):524‐530. doi:10.1089/dia.2013.0264 24455985

[41] Lou M , Luo P , Tang R , et al. Relationship between neutrophil‐lymphocyte ratio and insulin resistance in newly diagnosed type 2 diabetes mellitus patients. BMC Endocr Disord. 2015;15(1):9. doi:10.1186/s12902-015-0002-9 25887236 PMC4357061

[42] Wang RT , Zhang JR , Li Y , Liu T , Yu KJ . Neutrophil‐lymphocyte ratio is associated with arterial stiffness in diabetic retinopathy in type 2 diabetes. J Diabetes Complicat. 2015;29(2):245‐249. doi:10.1016/j.jdiacomp.2014.11.006 25483847

[43] Kahraman C . Neutrophil ‐ to ‐ lymphocyte ratio in diabetes mellitus patients with and without diabetic foot ulcer. Eur J Med Sci. 2014;1(1):8‐13. doi:10.12973/ejms.2014.102p

[44] Li X , Shen J , Lu Z , Chen M , Fang X , Wang G . High neutrophil‐to‐lymphocyte ratio is associated with increased carotid artery intima‐media thickness in type 2 diabetes. J Diabetes Investig. 2017;8(1):101‐107. doi:10.1111/jdi.12541 PMC5217917 27220111

[45] Sefil F , Ulutas KT , Dokuyucu R , et al. Investigation of neutrophil lymphocyte ratio and blood glucose regulation in patients with type 2 diabetes mellitus. J Int Med Res. 2014;42(2):581‐588. doi:10.1177/0300060513516944 24567354

[46] Oh Y , Kwon GC , Koo SH , Kim J . Association between glycemic control and hematologic indices in type 2 diabetic patients. Lab Med Online. 2016;6(3):134. doi:10.3343/lmo.2016.6.3.134

[47] Akin S , Aydin Z , Yilmaz G , Aliustaoglu M , Keskin O . Evaluation of the relationship between Glycaemic regulation parameters and neutrophil‐to‐lymphocyte ratio in type 2 diabetic patients. EMJ Diabetes. 2019;7(1):91‐96. doi:10.33590/emjdiabet/10311581

[48] Sheth J , Shah A , Sheth F , et al. The association of dyslipidemia and obesity with glycated hemoglobin. Clin Diabetes Endocrinol. 2015;1(1):6. doi:10.1186/s40842-015-0004-6 28702225 PMC5469195

[49] Duman TT , Aktas G , Atak BM , Kocak MZ , Erkus E , Savli H . Neutrophil to lymphocyte ratio as an indicative of diabetic control level in type 2 diabetes mellitus. Afr Health Sci. 2019;19(1):1602‐1606. doi:10.4314/ahs.v19i1.35 31148989 PMC6531946

[50] Huang Z , Xu Z , Xu R , Huang L , Xu X , Lai X . Whole exome sequencing identifies three novel gene mutations in patients with the triad of diabetic ketoacidosis, hypertriglyceridemia, and acute pancreatitis. J Diabetes. 2021;13(3):200‐210. doi:10.1111/1753-0407.13100 32734598

[51] Pezhman L , Tahrani A , Chimen M . Dysregulation of leukocyte trafficking in type 2 diabetes: mechanisms and potential therapeutic avenues. Front Cell Dev Biol. 2021;9:624184. doi:10.3389/fcell.2021.624184 33692997 PMC7937619

[52] Munteanu C , Rotariu M , Turnea MA , et al. Topical reappraisal of molecular pharmacological approaches to endothelial dysfunction in diabetes mellitus angiopathy. Curr Issues Mol Biol. 2022;44(8):3378‐3397. doi:10.3390/cimb44080233 36005129 PMC9406839

[53] Ushakumari CJ , Zhou QL , Wang YH , et al. Neutrophil elastase increases vascular permeability and leukocyte transmigration in cultured endothelial cells and obese mice. Cells. 2022;11(15):2288. doi:10.3390/cells11152288 35892585 PMC9332277

[54] Watanabe M . Risk factors and molecular mechanisms of esophageal cancer: differences between the histologic subtype. J Cancer Metastasis Treat. 2015;1:1‐7. doi:10.4103/2394-4722.153534

[55] Xia C , Rao X , Zhong J . Role of T lymphocytes in type 2 diabetes and diabetes‐associated inflammation. J Diabetes Res. 2017;2017:6494795. doi:10.1155/2017/6494795 28251163 PMC5307004

[56] Goldmann O , Nwofor OV , Chen Q , Medina E . Mechanisms underlying immunosuppression by regulatory cells. Front Immunol. 2024;15:1328193. doi:10.3389/fimmu.2024.1328193 38380317 PMC10876998

[57] Zhou T , Hu Z , Yang S , Sun L , Yu Z , Wang G . Role of adaptive and innate immunity in type 2 diabetes mellitus. J Diabetes Res. 2018;2018:7457269. doi:10.1155/2018/7457269 30533447 PMC6250017

[58] Yu W , Li C , Zhang D , et al. Advances in T cells based on inflammation in metabolic diseases. Cells. 2022;11(22):3554. doi:10.3390/cells11223554 36428983 PMC9688178

[59] Kullo IJ , Hensrud DD , Allison TG . Comparison of numbers of circulating blood monocytes in men grouped by body mass index (<25, 25 to <30, ≥30). Am J Cardiol. 2002;89(12):1441‐1443. doi:10.1016/S0002-9149(02)02366-4 12062747

[60] Huang Q , Wu H , Wo M , Ma J , Song Y , Fei X . Clinical and predictive significance of plasma fibrinogen concentrations combined monocyte‐lymphocyte ratio in patients with diabetic retinopathy. Int J Med Sci. 2021;18(6):1390‐1398. doi:10.7150/ijms.51533 33628095 PMC7893560

[61] Grossmannm V , Schmitt VH , Zeller T , et al. Profile of the immune and inflammatory response in individuals with prediabetes and type 2 diabetes. Diabetes Care. 2015;38(7):1356‐1364. doi:10.2337/dc14-3008 25877811

[62] Tanaka SI , Isoda F , Ishihara Y , Kimura M , Yamakawa T . T lymphopaenia in relation to body mass index and TNF‐α in human obesity: adequate weight reduction can be corrective. Clin Endocrinol. 2001;54(3):347‐354. doi:10.1046/j.1365-2265.2001.1139/cn2155.x 11298087

[63] Wellen KE , Hotamisligil GS . Inflammation, stress, and diabetes. J Clin Invest. 2005;115(5):1111‐1119. doi:10.1172/JCI200525102 15864338 PMC1087185

[64] Xue Y , Bao W , Huang W , Zou X , Guo Y . Relationship between neutrophil‐to‐lymphocyte ratio, monocyte‐to‐lymphocyte ratio, platelet‐to‐lymphocyte ratio and osteoporosis in postmenopausal type 2 diabetic patients: a retrospective study. Fortschr Med. 2024;103(50):e40869. doi:10.1097/MD.0000000000040869 PMC11651493 39686432

[65] Vaibhav NSS , Dongare D , Tripathi ACP , Tripathi T , Tripathi P . Deciphering the intricacies of immune system dysfunction and its impact on diabetes mellitus: revisiting the communication strategies to manage diabetes mellitus. Heal Sci Rev. 2024;13:100201. doi:10.1016/j.hsr.2024.100201

[66] Franceschi C , Capri M , Monti D , et al. Inflammaging and anti‐inflammaging: a systemic perspective on aging and longevity emerged from studies in humans. Mech Ageing Dev. 2007;128(1):92‐105. doi:10.1016/j.mad.2006.11.016 17116321

[67] Dayama N , Yadav SK , Saxena P , Sharma A , Kashnia R , Sharda K . A study of relationships between the HbA1c level and inflammatory markers, neutrophil‐to‐lymphocyte ratio, and monocyte‐to‐lymphocyte ratio in controlled and uncontrolled type 2 diabetes mellitus. J Assoc Physicians India. 2024;72(3):24‐26. doi:10.59556/japi.72.0427 38736112

[68] Gallagher EJ , Le Roith D , Bloomgarden Z . Review of hemoglobin a(1c) in the management of diabetes. J Diabetes. 2009;1(1):9‐17. doi:10.1111/j.1753-0407.2009.00009.x 20923515

[69] Glycemic targets: standards of medical care in diabetes−2021. Diabetes Care. 2021;44:S73‐S84. doi:10.2337/dc21-S006 33298417

[70] Petrillo T , Semprini E , Tomatis V , et al. Putative complementary compounds to counteract insulin‐resistance in PCOS patients. Biomedicine. 2022;10(8):1924. doi:10.3390/biomedicines10081924 PMC9406066 36009471

[71] Kautzky‐Willer A , Leutner M , Harreiter J . Sex differences in type 2 diabetes. Diabetologia. 2023;66(6):986‐1002. doi:10.1007/s00125-023-05891-x 36897358 PMC10163139

[72] Ingrosso DMF , Primavera M , Samvelyan S , Tagi VM , Chiarelli F . Stress and diabetes mellitus: pathogenetic mechanisms and clinical outcome. Horm Res Paediatr. 2023;96(1):34‐43. doi:10.1159/000522431 35124671

